# Complex interplay of interatomic bonding in a multi-component pyrophosphate crystal: K_2_Mg (H_2_P_2_O_7_)_2_·2H_2_O

**DOI:** 10.1098/rsos.170982

**Published:** 2017-12-06

**Authors:** Puja Adhikari, Redouane Khaoulaf, Hamid Ez-Zahraouy, Wai-Yim Ching

**Affiliations:** 1 Department of Physics and Astronomy, University of Missouri Kansas City, Kansas City, MO 64110, USA; 2 Department of Physics, Laboratory of Optoelectronics and Physical Chemistry of Materials, Faculty of Sciences, University lbn Tofail, Kenitra, Morocco; 3 Laboratory of Condensed Matter and Interdisciplinary Sciences (LAMCSCI), Faculty of Sciences, University Mohammed V, Rabat, Morocco

**Keywords:** interatomic bonding, electronic properties, pyrophosphates

## Abstract

The electronic structure and interatomic bonding of pyrophosphate crystal K_2_Mg (H_2_P_2_O_7_)_2_·2H_2_O are investigated for the first time showing complex interplay of different types of bindings. The existing structure from single-crystal X-ray diffraction is not sufficiently refined, resulting in unrealistic short O─H bonds which is rectified by high-precision density functional theory (DFT) calculation. K_2_Mg (H_2_P_2_O_7_)_2_·2H_2_O has a direct gap of 5.22 eV and a small electron effective mass of 0.14 *m*_e_. Detailed bond analysis between every pair of atoms reveals the complexity of various covalent, ionic, hydrogen bonding and bridging bonding and their sensitive dependence on structural differences. The K--O bonds are much weaker than Mg--O bonds and contributions from the hydrogen bonds are non-negligible. Quantitative analysis of internal cohesion in terms of total bond order density and partial bond order density divulges the relative importance of different types of bonding. The calculated optical absorptions show multiple peaks and a sharp Plasmon peak at 23 eV and a refractive index of 1.44. The elastic and mechanical properties show features unique to this low-symmetry crystal. Phonon calculation gives vibrational frequencies in agreement with reported Raman spectrum. These results provide new insights indicating that acidic pyrophosphates could have a variety of unrealized applications in advanced technology.

## 1. Introduction

Phosphates is a vast and complex subject with a long history intersecting multiple disciplines of physics, chemistry and biology. However, it is still not fully understood compared to another type of well-known inorganic crystals, the silicates [1]. The first research on phosphates can be traced to two centuries ago with the preparation of Na_4_P_2_O_7_ [2]. The vastly different classifications and overlapping nomenclatures of phosphates without consensus across different communities cause great difficulties and confusion among researchers. It is generally agreed that it originated with phosphoric acid H_3_PO_4,_ and the P atom is pentavalently bonded to four O atoms in forming a tetrahedral [PO_4_]^−3^ unit. Within this context, condensed phosphates can be described as compounds formed with cations consisting of alkali or other metallic elements and phosphoric anions denoted by (P*_n_*O_3*n*+1_)^−(*n*+2)^ (*n* < 20) or their various combinations with or without the presence of water molecules [1].

Some of the most common and well-known condensed phosphates include aluminium phosphate (AlPO_4_), potassium titanyl phosphate (KTP, KTiOPO_4_), calcium pyrophosphate dihydrate ((Ca_2_P_2_O_7_)·2H_2_O), hydroxyapatite (HAP, Ca_10_(PO_4_)_6_(OH)_2_), florapatite (FAP, Ca_10_(PO_4_)_6_F_2_) and amorphous calcium phosphates, adenosine triphosphate (ATP), lithium iron phosphates (LiFePO_7_), potassium dihydrogen phosphate (KDP, KH_2_PO_4_) and many more. These and other crystalline phosphates have many slightly different designations based on phosphorus content or the O/P ratio such as polyphosphate, pyrophosphate, metaphosphate, diphosphate, triphosphate, oligophosphate, cyclophosphate, etc. [1]. Pyrophosphate, also called diphosphate or dipolyphosphate, is the main subset of this large family of crystals originating from pyro phosphoric acid P_2_O_5_·2H_2_O (H_4_P_2_O_7_) which contains the pentoxide (P_2_O_5_) group.

Biologists believe that Nature chooses phosphates to be part of the genetic material such as DNA, RNA and ATP because phosphoric acid can meet the stringent condition required for the formation of linkage via hydrolytically stable ester bonds [3]. This leads to the assertion of evolutionary centrality of phosphates [3], as pointed out by Todd that ‘Where there is life, there is phosphorus' [4]. Organic phosphates and phosphate-based glasses are also an important part of biomaterials which is another extremely active arena of research [5,6]. There are essentially two types of bio-glasses: (i) a network glass such as ternary phosphate glass (CaO)_x_(Na_2_O)_1−x−y_(P_2_O_5_)y in the form of a continuous random network similar to silicate glasses and (ii) aqueous solution of orthophosphate species H*_n_*(PO_4_)^3−*n*^ (*n* = 0–3) important for biological reactions. Thus phosphates play an important role in biomedical and life sciences.

Applications of phosphates are ubiquitous because of their diverse compositions and unique complex structures. There are many biomedical and biochemical applications such as special drugs and their deliveries [7], bone repair, tissue engineering [8] and periodontal ligament fibroblasts for cell regeneration [9], etc. In energy science, they are known as predominating materials for Li or Na ion batteries [10,11]. They are also used in a variety of sensors [12,13] and in water oxidation catalysts [14]. Some phosphates such as KTP are known for their exceptionally large second harmonic generation for special electro-optical applications [15,16]. Hence, phosphates are critical to the development of modern technology.

In modern materials research, computational study always accompanies experimentation in any class of materials, and phosphates are no exception. A limited amount of computational works were done in tri- and diphosphates by Hansia *et al*. [17], interaction of pyrophosphate with hydroxyapatite by Rivas *et al*. [18] and cluster model studies on vanadyl pyrophosphates [19]. However, most of the studies in the past concentrate on the structures and geometry with little insights on the fundamental interactions at the atomic level and seldom touch on their properties which are important in their existing and emerging applications. This actually does not come as a surprise, given the confusing status of their definitions and many unsolved issues such as the accuracy of the measured structure, and the appropriate methods for different and diverse structures of phosphate. Most of the computational studies focus on molecular clusters rather than crystalline band structures. They also concentrate more on the vibrational spectra rather than the electronic structure. Quantitative information on different types of bonding in multi-component phosphates are missing. It turns out that we actually have several studies on different types of phosphates within the past decade with the earlier version of the methods we developed. They cover inorganic phosphates [20–22], apatite crystals and their spectroscopic or mechanical properties [23–25], and some major phosphor-olivines Li_x_MPO_4_ [26,27]. More recently, calculation has been extended to complex biomolecular systems containing phosphorus [28–32].

In this study, we focus on a particular acidic pyrophosphate crystal K_2_Mg (H_2_P_2_O_7_)_2_·2H_2_O because of its well-characterized crystal structure and detailed vibrational analysis on Raman and infrared spectra first reported by Harcharras *et al*. [33]. To the best of our knowledge, there have not been any electronic structure information for this crystalate or similar pyrophosphates. Here, we report the first such calculation with detailed interatomic bonding together with its optical and mechanical properties. Moreover, we show that, for such crystals, the experimentally determined structure may not be sufficiently accurate due to the difficulty in locating exact positions of H. We first describe its complex structure followed by a brief description of the computational methods used. The calculated electronic structure and properties are presented and discussed in §4. We end with a brief conclusion and discussion on the future prospect of research and potential applications of pyrophosphates in general.

## 2. Crystal structure of K_2_Mg (H_2_P_2_O_7_)_2_·2H_2_O

**Figure 1.**
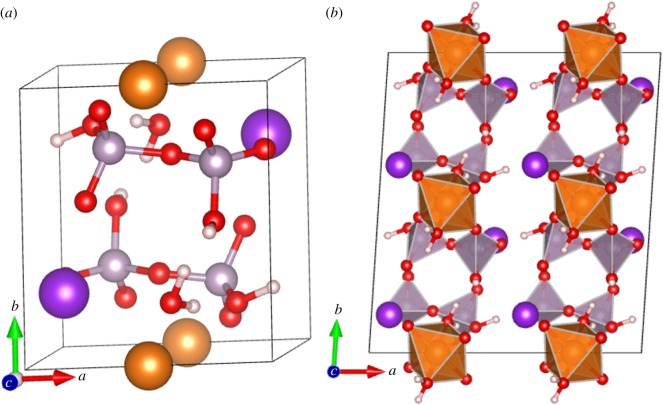
(*a*) Ball and stick diagram for the relaxed cell of K_2_Mg (H_2_P_2_O_7_)_2_·2H_2_O; (*b*) polyhedral diagram for the 2 × 2 × 2 supercell projected along the *c*-axis. MgO_6_ octahedra and PO_4_ tetrahedra are shown in orange and light purple, respectively. Dark purple sphere for K, small red sphere for O and small pinkish white sphere for H.

The chemical composition of all pyrophosphates contains the ionic group ${\text{P}}_{2}{{\text{O}}_{7}}^{4-}$. Here, we focus on KMg_0.5_H_2_P_2_O_7_·H_2_O (or K_2_Mg (H_2_P_2_O_7_)_2_·2H_2_O). This structure belongs to the di-cationic ABP_y_·nH_2_O family where A is a monovalent cation, B can be a mono-, di- or trivalent cation, and *P_y_* is the acidic pyrophosphate group (H_2_P_2_O_7_)^2−^. The crystal has a triclinic structure with space group *P_−_*_1_(2), *Z* = 2 with a total of 31 atoms in the unit cell as shown in figure 1*a*. The original synthesis of this pyrophosphate was done by Harcharras *et al*. [33] in 2003 and the single-crystal structure was obtained using X-ray diffractometry (XRD). The unit cell refinement used 1055 of the 1446 reflections observed by omitting those with intensity *I* less than 2*σ*/(*I*_obs_). The final structure was resolved using the direct method of SIR97 [34] and refined by a full-matrix least square technique based on F2-SHELXL-97 [35] with residue *R* = 0.0368 and supposed to be extremely accurate. Four years later, one of us (R.K.) used a new synthesis method, the co-precipitation method according to the reaction scheme: 2 K_4_P_2_O_7_ + MgCl_2_ + 4HCl to give K_2_Mg (H_2_P_2_O_7_)_2_·2H_2_O + 6KCl (dissolved). The recovered single crystal was then analysed in the same laboratory using the same high-precision procedure. The structure differs only slightly from the one published earlier by Harcharras *et al*. [33] (see electronic supplementary material for comparison). This structure is now referred to as the experiment XRD or unrelaxed structure in the present discussion. Figure 1*b* shows the polyhedral picture of this crystal in a 2 × 2 × 2 supercell showing layers of MgO_6_ octahedra and PO_4_ tetrahedra, and the locations of the K ions.

However, it was observed that there are two very short O─H bonds of 0.636 Å, 0.650 Å in the experimentally measured data which appear to be unrealistic. We proceed to investigate this problem using density functional theory (DFT) by firstly optimizing the measured structure theoretically using VASP (Vienna *ab initio* simulation package) [36]. We used PAW (projector augmented wave) with PBE potential [37] for the exchange and correlation functional within the generalized gradient approximation of the DFT. To achieve high accuracy for the structure optimization, a high cut-off energy of 600 eV is used. The electronic convergence and ionic force convergence are set at of 10^–9^ eV and 10^–7^ eV Å^−1^, respectively. A 3 × 3 × 3 *k*-point sampling using the standard Monkhorst scheme [38] as implemented in VASP is adopted. Test calculations by using other exchange correlation functionals such as hybrid functionals PBE0, HSE03 and also applying van der Waals correction show only very minor change in the relaxed structure.

**Figure 2.**
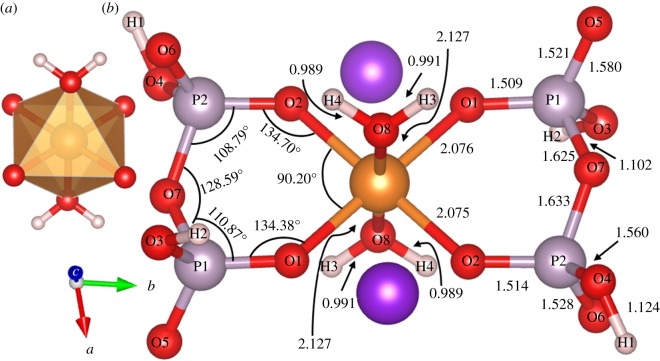
(*a*) Sketch of Mg-centred octahedron. (*b*) Two-dimensional sketch of Mg-centred K_2_Mg (H_2_P_2_O_7_)_2_·2H_2_O in the plane of diphosphate. The bond lengths shown are in units of Å. Large orange (dark purple) sphere for Mg (K), light purple sphere for P, small red sphere for O and small pinkish white sphere for H.

In table 1, we compare the experimental structure (XRD) and the final optimized structure (DFT) for K_2_Mg (H_2_P_2_O_7_)_2_·2H_2_O. It can be seen that there are some notable changes in the lattice constants with a slight increase in cell volume by 3.35%. Most importantly, the two unrealistically short O─H bonds of 0.636 Å and 0.656 Å are increased to a reasonable value of 1.102 Å and 1.124 Å, respectively. Simultaneously, the two hydrogen bonds (HBs), O···H, with bond lengths (BLs) 1.781 Å and 1.821 Å decreased to 1.304 Å and 1.349 Å of bridging bonds (i.e. O─H bonds from O─H─O, shown in figure 7 in blue circles), respectively. Another pair of HBs with BLs of 1.897 Å and 1.906 Å are also decreased to 1.807 Å and 1.768 Å, respectively. These changes have significant consequence in the subsequent analysis of interatomic bonding in this crystal (see §4.4). The total energy of the relaxed crystal is 19.12 eV lower than in the unrelaxed structure, or 59.51 KJ mol^−1^ per atom, indicating that the relaxed structure is more stable than the unrelaxed one. All different bond lengths for all atomic pairs are listed in table 1 for direct one-to-one comparison with those O─H, O···H and O─H─O bridging bonds with significant changes mentioned above shown in bold. Figure 2 shows a two-dimensional sketch of the relaxed structure with different bond angles and bond lengths clearly marked. In the MgO_6_ octahedron, Mg forms ionic bonds with four O atoms from the four PO_4_ tetrahedra and two O atoms from the two water molecules. In the two PO_4_ tetrahedra within the (H_2_P_2_O_7_)^2−^ group, one of its O atoms (O7) is bonded with another P atom in forming the strong P─O─P bond, which has been the centre of discussion in many pyrophosphate crystals and molecules. The two six-member rings with bonds P2─O7─P1─O1─Mg─O2─P2 and P1─O7─P2─O2─Mg─O1─P1 in figure 2 are symmetric with same bond lengths and bond angles. Ostensibly, some of the K─O separations are quite large (greater than 3.0 Å) and the formation of a K-centred polyhedron of these crystals is unlikely.

**Table 1.** Comparison between experimental XRD- and DFT-relaxed structures.

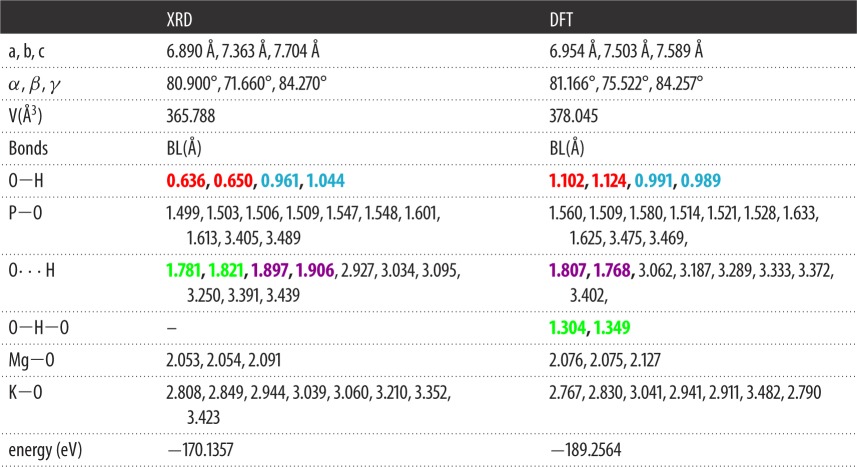

## 3. Method of electronic structure and properties calculation

For the electronic structure, interatomic bonding and optical properties of the titled crystal, we use the Orthogonalized linear combination of atomic orbital (OLCAO) method [39], which is also a DFT-based method. The VASP-optimized structure described above is used as input for the OLCAO calculation. The OLCAO method uses atomic orbitals for the basis expansion and is very efficient as well as economical, especially for large complex systems. It has been used previously to study the electronic structure and spectroscopic properties of many phosphate-related inorganic crystals and their surfaces [21–27]. The use of the OLCAO method with a VASP-optimized structure has resulted in many successful calculations in electronic and optical properties for large complex materials including inorganic glasses [40–44] and biomolecules [30–32].

In most OLCAO calculations, a more localized minimal basis (MB) is used for the calculation of effective charges $Q_{\alpha }^{*}$ and the bond-order (BO) values *ρ_αβ_* between a pair of atoms (*α*, *β*) using Mulliken population analysis [45,46].

3.1 $$Q_{\alpha }^{*}=\underset{i}{\sum }\underset{m,\text{occ}}{\sum }\underset{j,\beta }{\sum }C_{i\alpha }^{*m}C_{j\beta }^{m}S_{i\alpha ,j\beta }$$

and

3.2 $$\rho_{\alpha \beta }=\underset{m.\text{occ}}{\sum }\underset{i,j}{\sum }C_{i\alpha }^{*m}C_{j\beta }^{m}S_{i\alpha ,j\beta }.$$

In equations (3.1) and (3.2), $S_{i\alpha ,j\beta }$ are the overlap integrals between the *i*th orbital in the *α*th atom and the *j*th orbital in the *β*th atom; and *C^m^_jβ_* are the eigenvector coefficients of the *m*th band, *j*th orbital in the *β*th atom. We have calculated the partial charge Δ*Q* or the charge transfer which is a deviation from neutral charge (*Q*^o^) of the effective charge (Q*) on the same atom i.e. Δ*Q* = *Q*^o ^– Q*. The MB set used in the present study has the atomic configurations of P: 1s^2^ 2s^2^ 2p^6^ 3s^2^ 3p^3^; Mg: 1s^2^ 2s^2^ 2p^6^ 3s^2^; K: 1s^2^ 2s^2^ 2p^6^ 3s^2^ 3p^6^ 3d 4s^1^; O: 1s^2^ 2s^2^ 2p^4^ and H: 1s^1^. A full-basis (FB) set which has one more shell of atomic orbitals than the MB is used for calculation of self-consistent potential, the total density of states (TDOS), partial density of states (PDOS), band structure and optical properties.

The bond order (BO) *ρ_αβ_* in equation (3.2) provides a quantitative measure for the strength of the bond. The summation of all BOs normalized by the cell volume gives us the total bond order density (TBOD), which is a single metric to assess the internal cohesion in the crystal [47]. TBOD can be resolved into partial components, or the partial BO density (PBOD) from different types of atomic pairs or groups of atoms in a structural unit.

For the interband optical properties in the form of frequency-dependent complex dielectric function (ℏω) = *ε*_1_(ℏω) + i*ε*_2_(ℏω), the imaginary part *ε*_2_(ℏω) is calculated first according to

3.3 $$\varepsilon_{2}(\hbar \omega )=\frac{e^{2}}{\pi n\omega^{2}}\int_{BZ}\text{d}k^{3}\underset{nl}{\sum }|\langle \psi_{n}(k,r)|-i\hbar \nabla |\psi_{l}(k,r)\rangle {|}^{2}f_{l}(k)[1-f_{n}(k)]\delta [E_{n}(k)-E_{l}(k)-\hbar \omega ],$$

where *l* and *n* are for the occupied and unoccupied states, respectively, $\psi_{n}(k,r)$ are the *ab initio* Bloch functions from OLCAO calculation using FB and a large *k*-point sampling, and *f*_1_(*k*) and *f_n_*(*k*) are the Fermi distribution functions. The real part $\varepsilon_{1}(\hbar \omega )$ is obtained from the imaginary part $\varepsilon_{2}(\hbar \omega )$ through Kramers–Kronig transformation [48]. The energy loss function, *F*(*ω*) is obtained from the imaginary part of (1/*ε*).

3.4 $$F(\omega )=\text{IM}\left(-\frac{1}{\varepsilon (\omega )}\right)=\frac{\varepsilon_{2}(\omega )}{\varepsilon_{1}^{2}(\omega )+\varepsilon_{2}^{2}(\omega )}.$$

The elastic and mechanical properties of the pyrophosphate crystal are calculated using the stress versus strain approach to obtain the complete set of elastic coefficient *C_ij_* and compliance tensor. The fully relaxed crystal structure from VASP is used to calculate the second-order elastic tensors for the crystal using an efficient scheme [49,50] by applying a strain of ±0.5% to the cell to obtain the stress data *σ_j_*. The elastic coefficients *C_ij_* are obtained by solving the set of linear equation

3.5 $$\sigma_{i}=\overset{6}{\underset{j=1}{\sum }}C_{ij}\varepsilon_{j}\;(i,j=1,2,3,4,5,6).$$

The usual mechanical properties *K* (bulk modulus), *G* (shear modulus), *E* (Young's modulus) and *η* (Poisson's ratio) are obtained based upon the Voigt–Reuss–Hill approximation for polycrystals [51,52].This approach for mechanical properties has been successfully used by us in many different inorganic crystals and glasses [44,53–57].

## 4. Results and discussion

### 4.1. Band structure and density of states

**Figure 3.**
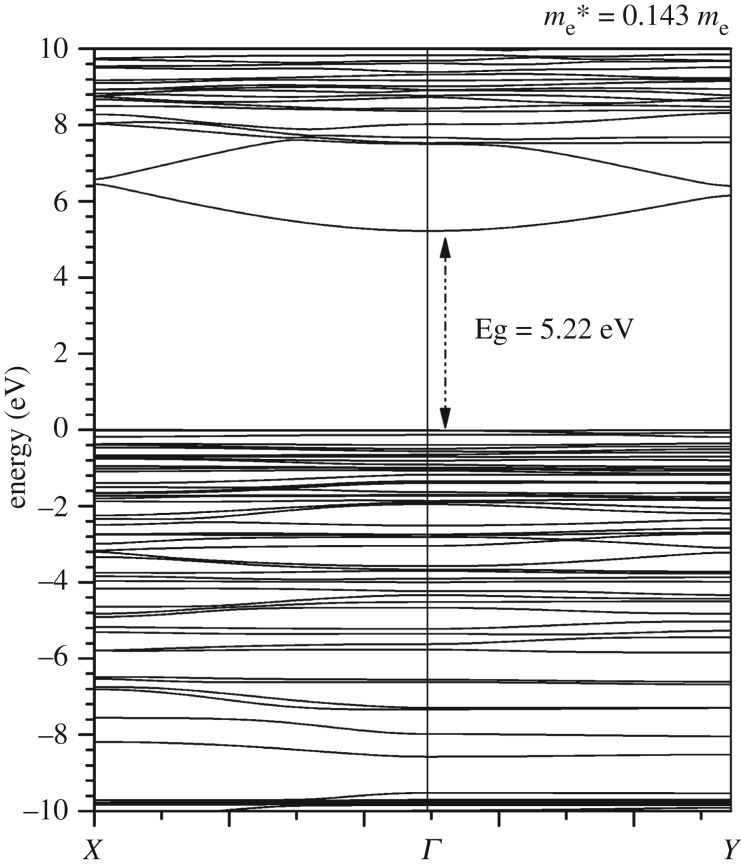
Calculated band structure for K_2_Mg (H_2_P_2_O_7_)_2_·2H_2_O. The electron effective mass at *Γ* is $m_{e}^{*}=0.143m_{e}.$

The calculated band structure for the triclinic K_2_Mg (H_2_P_2_O_7_)_2_·2H_2_O crystal is shown in figure 3. It has a direct band gap (Eg) at *Γ* of 5.22 eV, larger than the other phosphate crystal HCP (4.51 eV) and smaller than FAP (5.47 eV) [23] calculated using the same method. The wide band gap shows the insulating nature of K_2_Mg (H_2_P_2_O_7_)_2_·2H_2_O. The top of the valence band (VB) is quite flat, whereas the bottom of the conduction band (CB) has a curved feature with an effective electron mass of *m*_e_* = 0.143 *m*_e_, comparable to the wide-gap semiconductors such as in AlP (0.13*m*_e_), GaN (0.19*m*_e_), ZnO (0.24*m*_e_), ZnSe (0.17*m*_e_) and ZnTe (0.18*m*_e_). This could imply that it will have large electron mobility in the CB with useful applications.

**Figure 4.**
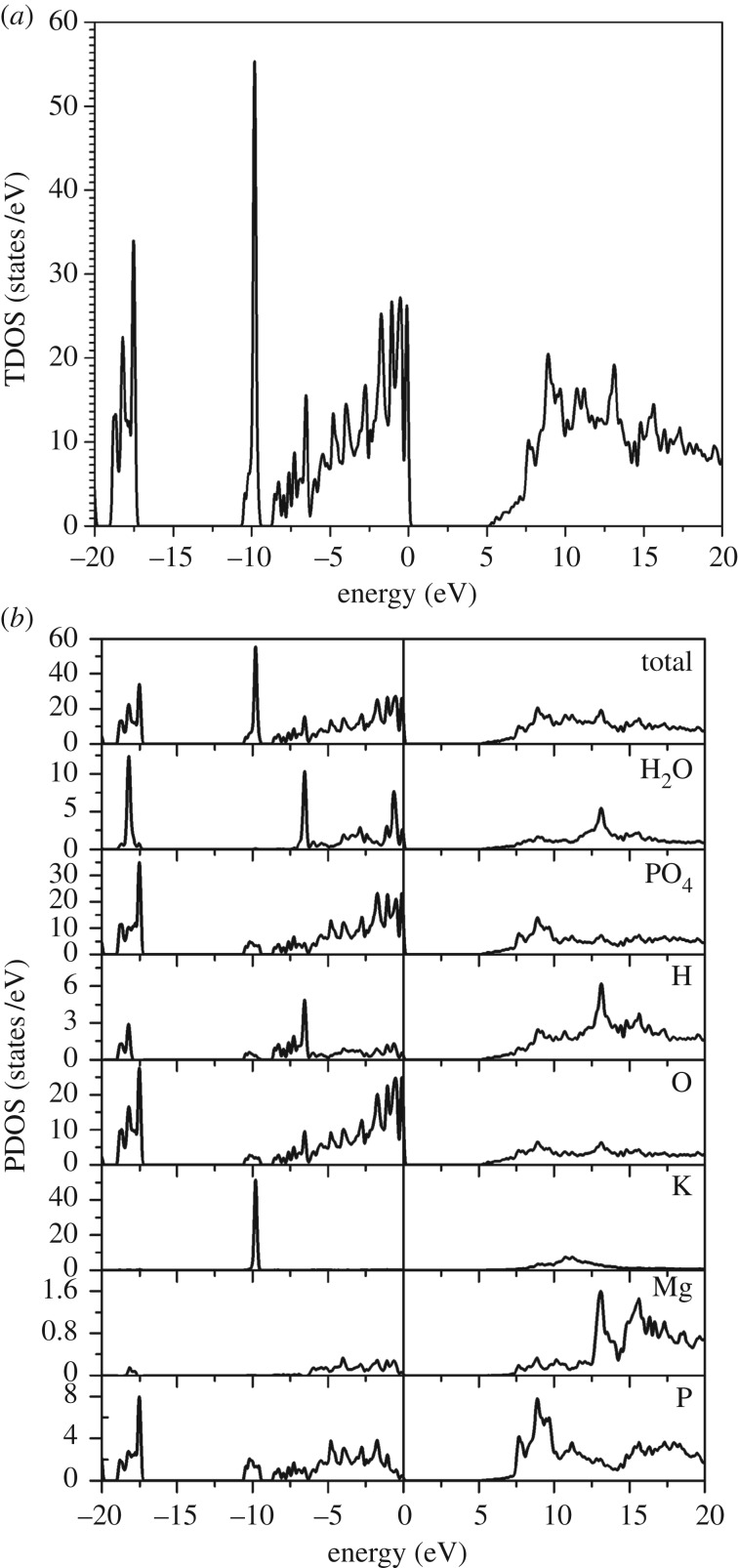
Calculated (*a*) total density of states (TDOS) and (*b*) partial density of states (PDOS) for H_2_O and PO_4_ groups and for each type of atom in K_2_Mg (H_2_P_2_O_7_)_2_·2H_2_O.

The calculated TDOS in the energy range of −20 to 20 eV is shown in figure 4*a*. Details of TDOS are best discussed in terms of the PDOS for each type of atoms and the two groups (H_2_O) and (PO_4_) shown in figure 4*b*. The peaks in the region lower than –15 eV come from P, O and H atoms, indicating the strong P─O bonding in the PO_4_ tetrahedron and O─H bonding in H_2_O. There is a very sharp peak at −10 eV from K-3p which simply reflects the semi-core nature of the K-3p orbital. All elements are responsible for the peaks in the energy range −10 to 0 eV, or the upper VB. The top of the VB is dominated by the states from PO_4_ tetrahedrons in the pyrophosphate group (H_2_P_2_O_7_)^−2^. Inspection of the wave function for the HOMO and LUMO states reveals that they come from the binding and anti-binding orbitals of the O─P bonds, which is a universal feature in almost all phosphates.

### 4.2. Partial charge

**Figure 5.**
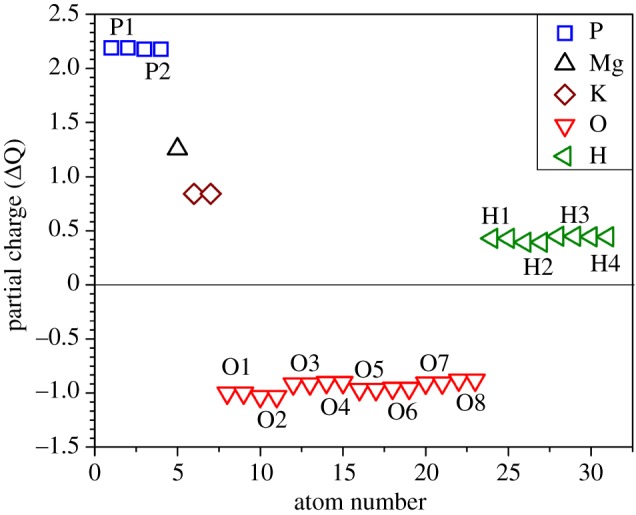
Calculated partial charge (Δ*Q*) distribution in K_2_Mg (H_2_P_2_O_7_)_2_·2H_2_O. The numbered atoms are in accordance with table 2.

The partial charges (PCs) obtained from the effective charges according to equation (3.1) for the 31 atoms in the K_2_Mg (H_2_P_2_O_7_)_2_·2H_2_O crystal are listed in table 2 and plotted in figure 5. All atoms except O have positive PCs that donate electrons to O. The largest positive PC is from P (+2.179 e and +2.189 e) in forming PO_4_ tetrahedra, which have negative charge as a unit. The divalent Mg ion has a larger PC (+1.256 e) than the monovalent K potassium (+0.842 e), and the PC for H atoms differ slightly (+0.393 e to +0.449 e) depending on the atoms they bond to. Note that the PC for the eight types of O are all different because they are crystallographically non-equivalent. This can be best seen in figure 2 where the positions of the labelled atoms in the sequential order are marked. Figure 5 shows that there are two data points each for O1 to O8. The O1 atoms form the P1─O1─Mg bonds connecting the PO_4_ tetrahedron to the MgO_6_ octahedron in the 6-member ring. Similarly, O2 form the other P2─O2─Mg bonds in the same 6-member ring. O3 and O4 form a P1─O3─H2 bond and P2─O4─H1 bond, respectively, involving the two elongated O─H bonds with BLs of 1.102 Å and 1.124 Å that has been emphasized in §2 above in the structure change due to optimization. O6 is the atom bonded only to P2, whereas O7 is forming the bridging bond between two phosphorus atoms P1 and P2. Finally, O8 is the atom in the two water molecules but they also participate in the formation of the octahedra centred at Mg. The above discussion on the PC distribution of all atoms within the unit cell of triclinic K_2_Mg (H_2_P_2_O_7_)_2_·2H_2_O crystal provided the most detailed and quantitative analysis of the charge transfer in the crystal in relation to their geometric arrangements.

**Table 2.** Different partial charge of each element in the DFT-relaxed structure.

element	Δ*Q*	element	Δ*Q*
P1	2.189	O1	−0.999
P2	2.179	O2	−1.031
Mg	1.256	O3	−0.914
K	0.842	O4	−0.902
H1	0.429	O5	−0.966
H2	0.393	O6	−0.955
H3	0.449	O7	−0.906
H4	0.444	O8	−0.880

### 4.3. Interatomic bonding

**Figure 6.**
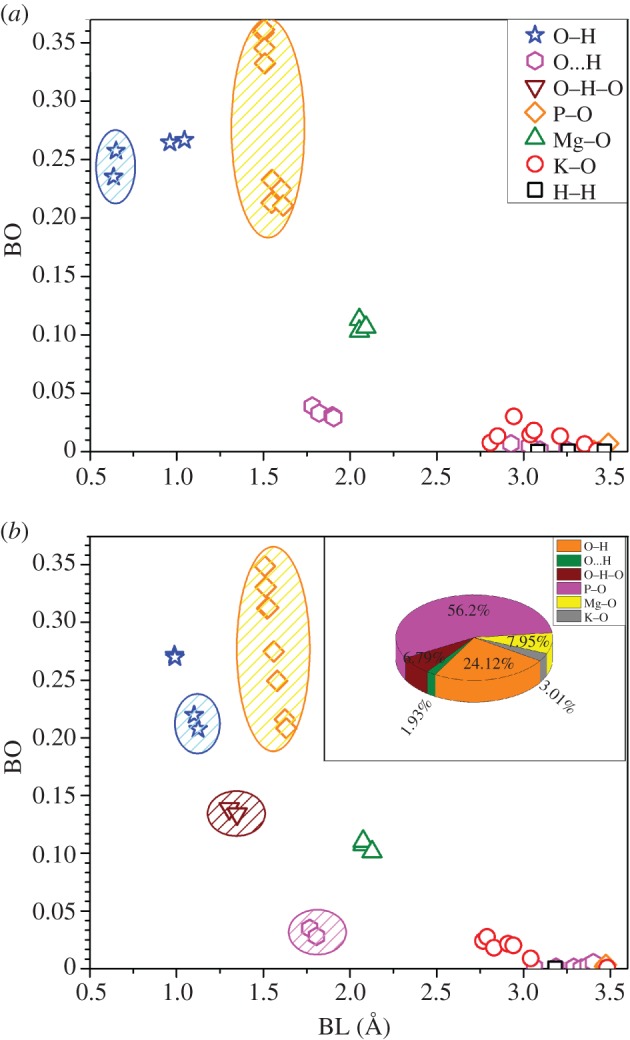
Calculated bond order (BO) versus bond length (BL) of K_2_Mg (H_2_P_2_O_7_)_2_·2H_2_O for (*a*) unrelaxed and (*b*) relaxed crystal. Each bond type is denoted by different symbols. Elliptical enclosures denote different groups of bond pairs (see text for details.) Inset: % contribution to the TBOD from different covalent, ionic and hydrogen bonds.

**Figure 7.**
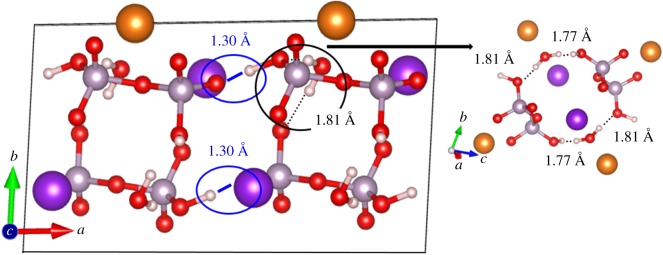
Illustration of the short and stronger bridging O─H─O bond of 1.30 Å (shown in blue) after optimization between two H_2_P_2_O_7_ groups in the adjacent cells (the lower bridging bond is not clear; there is an oxygen atom behind a potassium atom). Dark purple sphere for K, small red sphere for O and small pinkish white sphere for H. The figure on the right shows O···H bonds of 1.77 Å and 1.81 Å.

The BO values are a measure of strength and stiffness of the bond for each atomic pair. The calculated BO distribution versus bond length (BL) for different pairs of bonds in K_2_Mg (H_2_P_2_O_7_)_2_·2H_2_O are displayed in figure 6, figure 6*a* for the results from the original experimental structure and figure 6*b* for the optimized or the final relaxed structure. There are seven different types of bonding with varying strengths: two covalent bonds (O─H, P─O), two ionic bonds (Mg─O, K─O), the ubiquitous HB (O···H), bridging bonds O─H─O (in optimized structure only) and negligibly weak H─H bonds. The two unusually short O─H bonds of BLs 0.63 Å and 0.64 Å with high BO values depicted in figure 6*a* have been corrected after optimization to 1.10 Å and 1.12 Å (O3─H2 and O4─H1 in figure 2) and reasonable BO values (figure 6*b*, blue ellipse). Another major change due to optimization is that, in the unrelaxed experimental structure, the four HBs (O···H) are relatively weak with typical HB length of 1.8 Å. After optimization, two of the HBs become much stronger with BO values of 0.139 and 0.134, and BLs of 1.304 Å and 1.349 Å, respectively, forming a bridging bond O─H─O (brown inverse triangle in figure 6*b*). The other two HBs remain similar (pink ellipse in figure 6*b*). These HB configurations are more clearly shown in figure 7 on the extended cell geometry. The change in the covalent P─O bonds between unrelaxed and optimized structures are much smaller, accentuating the rigidity of the PO_4_ tetrahedron as a strong structural unit in phosphate crystals. The minor changes in the spread of BO values can be attributed to structural changes of the more prominent changes in O─H bonds, HBs and bridging bonds. As for the ionic bonds, the changes due to optimization is minimal. Mg─O bonds are relatively stronger than the K─O bonds and they do form the octahedral unit. K─O bonds are very weak and they cannot be considered to form any type of polyhedrons in phosphates at least in the present diphosphate crystal but may do so in some non-crystalline phosphate bio-glasses [33].

TBOD is the sum of total bond order in the system normalized by volume. We use the TBOD to assess the cohesion in the system. Table 3 compares the TBO and TBOD and their components, the PBOD for the unrelaxed and the optimized structure. As can be seen, the TBO in the optimized structure is higher than that in the original structure by 3.4%, consistent with the total energy shown in table 1. The inset of figure 6*b* shows the percentage contribution from a covalent bond, ionic bond and HB in terms of the PBOD. The P─O bonding has a major contribution of 56.2% in the system and provides the main cohesion in the crystal. Thus the P─O bonds are the most important bonding in the formation of the pyrophosphate. This is the main reason that pyrophosphates are the key structural unit in almost all inorganic phosphates and in forming the backbone in biological or genetic materials. On the other hand, O─H bonds (24.12%) also have significant cohesion for the system. Bridging O─H─O bonds (6.79%) and Mg─O (7.95%) bonds have a similar contribution. However, O···H (1.93%), K─O (3.01%) bonds have an insignificant contribution to the cohesion in this system. The comparison with a similar calculation of BO values with the other experimental structure from Harcharras *et al*. [33] is shown in electronic supplementary material, table S1. It is quite obvious that the optimized structure has the largest TBO and TBOD indicating a stronger crystal cohesion.

**Table 3.** Comparison between TBOD and PBOD using experimental and DFT-relaxed structures.

bonds	PBOD (XRD) (electron/(Å)^3^)	PBOD (DFT) (electron/(Å)^3^)
O─H	0.00560	0.00512
O⋯H	0.00081	0.00041
O─H─O	–	0.00144
P─O	0.01250	0.01194
Mg─O	0.00176	0.00169
K─O	0.00057	0.00064
TBOD	0.02124	0.02125
TBO (e^−^)	7.76920	8.03360

### 4.4. Optical properties and refractive index

**Figure 8.**
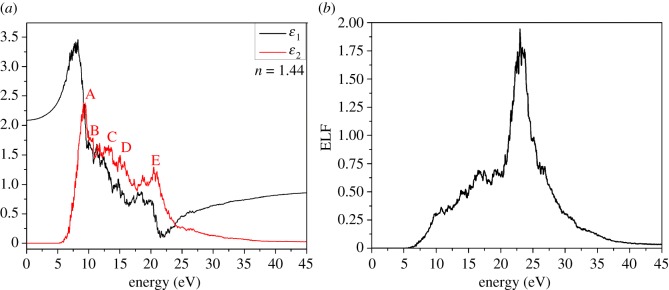
(*a*) Calculated real (*ε*_1_) (black curve) and imaginary (*ε*_2_) (red curve) parts of the complex dielectric function and (*b*) energy loss function (ELF) for K_2_Mg (H_2_P_2_O_7_)_2_·2H_2_O.

The optical properties of K_2_Mg (H_2_P_2_O_7_)_2_·2H_2_O are calculated in the form of complex dielectric function based on interband optical transitions. Figure 8*a* shows the calculated real (*ε*_1_) and imaginary (*ε*_2_) parts of frequency-dependent dielectric function in black and red lines. The optical absorption spectrum (*ε*_2_) shows five well-defined peaks A, B, C, D, E roughly at 7, 10, 13, 16, 21 eV, with peaks A and E the most prominent. These absorptions occur in the vacuum ultraviolet and part of the extreme ultraviolet region, well beyond the visible range and consistent with the transparent nature of the crystal. From the calculated dielectric functions, we can obtain the static dielectric constant by taking the zero-frequency limit of the real part of the dielectric function. The refractive index is estimated by taking the square root of *ε*_1_(0). For K_2_Mg (H_2_P_2_O_7_)_2_·2H_2_O, the calculated refractive index is 1.44. This can be compared with refractive indices of some common materials such as 1.33 for water, 1.458 for fused silica, 1.44–1.47 for olive oil and 1.42 for the 50% sugar solution. Hence, it is conceivable that dicationic pyrophosphates and other related crystalline or amorphous phosphates can find potential applications in technology similar to those of the silicate glasses or in biological fluids.

Figure 8*b* shows the energy loss function (ELF) obtained from the complex dielectric function. The main peak in ELF is identified as the plasma frequency (*ω*_p_) and is 22.98 eV. This is the frequency for the collective excitation of the electrons in a solid and is experimentally an easily measurable quantity. Thus, calculation of optical properties of different pyrophosphates can be effectively used as a predictor for the possible phases in an unknown sample.

### 4.5. Mechanical properties

As pyrophosphates may have many diversified uses, it is essential to know their mechanical properties like bulk modulus (*K*), shear modulus (*G*), Young's modulus (*E*) and Poisson's ratio (*η*). Table 4 lists the calculated elastic tensor *C_ij_* from equation (3.5). K_2_Mg (H_2_P_2_O_7_)_2_·2H_2_O has a triclinic structure of very low symmetry, so all the 22 second-order tensor elements are non-zero. The largest one is *C*_11_ followed by *C*_22_ and *C*_33,_ which are very close. It was noted that the off-diagonal element *C*_13_ is actually negative but small, which could be related to the rather complex structure of K_2_Mg (H_2_P_2_O_7_)_2_·2H_2_O, with the presence of water molecules discussed in §2. The calculated *K* and *G* are 22.88 GPa and 15.63 GPa, respectively. The low *K* and *G* show K_2_Mg (H_2_P_2_O_7_)_2_·2H_2_O with a *G*/*K* ratio of 0.691, which shows the materials to be brittle and not soft. This seems to be counterintuitive to the fact that K_2_Mg (H_2_P_2_O_7_)_2_·2H_2_O contains water molecules and a larger number of weak ionic bonds. The calculated *E* is 38.19 GPa and the Poisson's ratio *η* is 0.2218. This value is smaller than similar calculations on HAP (0.262) [58] which contains the unlinked PO_4_ tetrahedrons. The low Poisson's ratio *η* and high *E* for K_2_Mg (H_2_P_2_O_7_)_2_·2H_2_O also suggest that the material is less ductile and stiff in nature. This could be linked to the rigidity of the pyrophosphate units in contrast to the isolated PO_4_ unit. This is the first time that the mechanical properties of a pyrophosphate crystal have been calculated and much deeper analysis of their intriguing behaviour needs further investigation. We are not aware of any experimental measurements on the mechanical properties of pyrophosphate crystals.

**Table 4.** Calculated elastic coefficients of K_2_Mg (H_2_P_2_O_7_)_2_·2H_2_O in units of GPa.

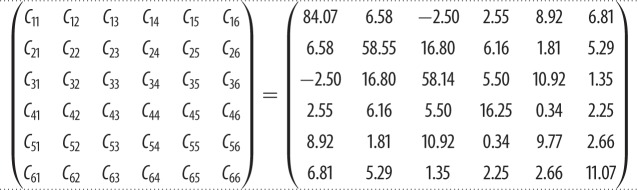

### 4.6. Connection to experimental vibrational spectrum

It is not clear at this moment if the above new findings may influence the interpretation of the vibrational properties because Raman and infrared measurements were conducted on actual samples and did not explicitly depend on the reported crystal parameters [33]. It would be of great interest to establish a connection between the electronic structure and bonding to the discussion of the vibrational spectra, which have many interesting features at the low-frequency region. To this end, we have calculated the phonon spectrum of the K_2_Mg (H_2_P_2_O_7_)_2_·2H_2_O crystal using the phonopy package [59], with VASP for the force calculation. This requires even higher accuracy because it involves a higher vibration frequency of O─H modes and the HBs. The convergence criteria for electronic and ionic force are now set at 10^−9^ eV and 10^−7^eV Å^−1^, respectively. The calculated phonon spectrum and phonon DOS are shown in electronic supplementary material, figure S1a,b, respectively. The vibrational frequencies at the zone centre *Γ* gives the information on Raman and infrared active modes. The vibrational frequency range can be divided into three regions I (0–37.72 THz), II (38.34–57 THz) and III (97.67–105.79 THz) (electronic supplementary material, figure S1b). This is in very good agreement with the measured Raman spectrum as shown in fig. 5 of Harcharras *et al.* [33], both showing multiple peak structures in region I, very pronounced double peak structure in region II and H-related vibration modes in region III separated from region II with a large gap.

## 5. Conclusion

In this article, we have presented a detailed analysis of the electronic structure, interatomic bonding, optical and mechanical properties calculation for the acidic diphosphate K_2_Mg (H_2_P_2_O_7_)_2_·2H_2_O. This is the first such study in this pyrophosphate crystal of very low symmetry. It is concluded that the existing crystal structure data from XRD is not sufficiently refined, which results in unrealistically short O─H bonds. This is rectified by high-precision computational optimization. The results show the insulating nature of this crystal with a large HOMO─LUMO gap of 5.22 eV. The P─O bond has a large bond order and the highest percentage contribution to the TBOD among all other bonds, indicating the high rigidity of the ${\left({\text{P}}_{2}{\text{O}}_{7}\right)}^{4-}$ ion in the pyrophosphate. The important role of strong O─H covalent bonds, substantial O─H─O bridging bonds and O⋯H HBs in this pyrophosphate is pointed out. It also shows that the formation of a K-centred polyhedron is unlikely due to the very weak K─O bonds. On the other hand, the Mg─O bonds are reasonably strong in forming the octahedral unit of MgO_6_ with two O atoms from the water molecules.

The most significant part of the above finding is that conventional XRD data and its structural analysis may be questionable for crystals involving light H atoms because of their weak intensity signals in the measurement. This could affect many of the published data on the structures of pyrophosphates. High-level DFT calculation can ameliorate the situation and should be part of future crystallographic analysis. The combination of an optical spectrum with large absorption at much higher energy beyond the ultraviolet region and the interesting mechanical properties unique to this low-symmetry crystal, and their possible connection to the low-frequency vibrational spectra offer new insights and opportunities for novel applications of the pyrophosphate materials.

We end this section with comments on possible extension of the present work and what specific aspects of the properties calculation can be related to real applications. Firstly, there are many similar diphosphate crystals such as K_2_Cu(H_2_P_2_O_7_)_2_·2H_2_O [60], (NH_4_)_2_Zn(H_2_P_2_O_7_)_2_·2H_2_O [61], (NH_4_)_2_Mn(H_2_P_2_O_7_)_2_·2H_2_O [62] and K_2_Zn(H_2_P_2_O_7_)_2_·2H_2_O [63], where the presence of transition metals could significantly affect its electronic structure and properties, leading to some unexpected applications. Secondly, the calculation can be extended to triphosphates or even to non-crystalline pyrophosphate glasses where the application in biological or medical areas are abundant. The OLCAO method is fully capable of treating such complex systems as demonstrated in many recent applications to large biomolecular systems [28,29,31,32] and inorganic glasses [41–43,56].

## Supplementary Material

Tables, Figures and structure data

## Data accessibility

The datasets supporting this article have been uploaded as part of the electronic supplementary material.
