# Supplementary material for: Complex interplay of interatomic bonding in a multi-component pyrophosphate crystal: K2Mg (H2P2O7)2·2H2O
Source: R Soc Open Sci. 2017 Dec 6;4(12):170982. doi: 10.1098/rsos.170982 (PMC5750006; doi:10.1098/rsos.170982)
Supplement: Tables, Figures and structure data [file rsos170982supp1.docx]

**Supplementary Material**

**Complex Interplay of Interatomic Bonding in a Multi-component Pyrophosphate Crystal: K_2_Mg (H_2_P_2_O_7_)_2_∙2H_2_O**

Puja Adhikari^a^, Redouane Khaoulaf^b,c^, Hamid Ez-Zahraouy^c^ and Wai-Yim Ching^a^

1. Department of Physics and Astronomy, University of Missouri Kansas City, Kansas City Missouri, 64110 USA
2. Department of Physics, Laboratory of Optoelectronics and Physical Chemistry of Materials, Faculty of Sciences, University lbn Tofail, Kenitra, Morocoo
3. Laboratory of Condensed Matter and Interdisciplinary Sciences, Faculty of Sciences, University Mohammed V Rabat, Morocco

Corresponding author: Wai-Yim Ching [Chingw@umkc.edu](mailto:Chingw@umkc.edu)

**Table S1:** Comparison of the 2 experimental structures. Current work and reference [33].

|  | **XRD ref. [33]** | **XRD [current work]** | **DFT** |
| --- | --- | --- | --- |
| **a, b, c** | 6.857Å, 7.362Å, 7.620Å | 6.890Å, 7.363Å, 7.704Å | 6.954Å, 7.503Å, 7.589Å |
| **α, β, γ** | 81.044Å, 72.248Å, 83.314Å | 80.900°, 71.660°, 84.270° | 81.166°, 75.522°, 84.257° |
| **V(Å^3^)** | 360.899 | 365.788 | 378.045 |
| **Bonds** | BL(Å) | BL(Å) | BL(Å) |
| **O–H** | 0.673, 0.805, 0.813, 0.953 | 0.636, 0.650, 0.961, 1.044 | 1.102, 1.124, 0.991, 0.989 |
| **P–O** | 1.492, 1.494, 1.504, 1.547,1.602, 1.611, 3.480 | 1.499, 1.503, 1.506, 1.509, 1.547, 1.548, 1.601, 1.613, 3.405, 3.489 | 1.560, 1.509, 1.580, 1.514, 1.521, 1.528, 1.633, 1.625, 3.475, 3.469 |
| **O⋯ H** | 1.588,1.858, 1.930, 2.004, 2.822, 2.940, 3.060, 3.083, 3.262, 3.303 | 1.781, 1.821, 1.897, 1.906, 2.927, 3.034, 3.095, 3.250, 3.391, 3.439 | 1.304, 1.349,1.807, 1.768, 3.062, 3.187, 3.289, 3.333, 3.372, 3.402 |
| **Mg–O** | 2.053, 2.059, 2.100 | 2.053, 2.054, 2.091 | 2.076, 2.075, 2.127 |
| **K–O** | 2.767, 2.807, 2.886, 2.886,2.972, 2.999, 3.235, 3.332, 3.421 | 2.808, 2.849, 2.944, 3.039, 3.060,3.210, 3.352, 3.423 | 2.767, 2.830, 3.041, 2.941, 2.911, 3.482, 2.790 |

Note: Here the XRD structure from ref. [33] also have shorter O-H bonds at 0.67Å.

**Table S2:** Comparison of the calculated total and partial bond order density from the two experimental structure and the relaxed structure.

| **Bonds** | **PBOD (XRD) ref-[33] electron/(Å)^3^)** | **PBOD (XRD) (electron/(Å)^3^)** | **PBOD (DFT) (electron/(Å)^3^)** |
| --- | --- | --- | --- |
| **O–H** | 0.00592 | 0.00560 | 0.00512 |
| **O⋯ H** | 0.00092 | 0.00081 | 0.00185 |
| **P–O** | 0.01267 | 0.01250 | 0.01194 |
| **Mg–O** | 0.00174 | 0.00176 | 0.00169 |
| **K–O** | 0.00059 | 0.00057 | 0.00064 |
| **TBOD** | 0.02183 | 0.02124 | 0.02125 |
| **TBO (e)** | 7.87970 | 7.76760 | 8.03360 |

**Fig. S1: (a)** The calculated phonon spectrum and **(b)** phonon DOS divided into three regions.

Relaxed structure of K_2_Mg (H_2_P_2_O_7_)_2_∙2H_2_O in the form of POSCAR from VASP package.

System P4MgK2O16H8

1.00000000000000

6.9470138460243573 -0.0501741998533689 -0.2984441001881987

0.8045681316646195 7.4597302340065150 -0.0190116836837915

2.2152604346030293 0.9517288504657606 7.1961218859002285

P Mg K O H

4 1 2 16 8

Direct

0.2542989361917557 0.3028673382233372 0.2626037566796885

0.7457010638082444 0.6971326617766623 0.7373962433203080

0.6813818648407474 0.2400490431462951 0.2467751943754752

0.3186181351592603 0.7599509568537063 0.7532248056245198

0.5000000000000000 -0.0000000000000000 0.0000000000000000

0.1301958785572582 0.2512378811273653 0.7863473994050708

0.8698041214427427 0.7487621188726375 0.2136526005949301

0.2744936662330049 0.1776074781939230 0.1204611531553422

0.7255063337669897 0.8223925218060780 0.8795388468446574

0.2521287075436682 0.5071418703042755 0.1724890741258545

0.7478712924563377 0.4928581296957274 0.8275109258741412

0.0827848532642706 0.2767707944710715 0.4318804516313530

0.9172151467357328 0.7232292055289213 0.5681195483686498

0.7652517495085563 0.4194434871536125 0.1490457726874619

0.2347482504914342 0.5805565128463929 0.8509542273125389

0.6968136322994365 0.0975326101184932 0.1218706200831292

0.3031863677005543 0.9024673898815082 0.8781293799168796

0.7701447267028384 0.1701607790797788 0.4161631618903360

0.2298552732971561 0.8298392209202391 0.5838368381096615

0.4478378420483854 0.2768373322345757 0.3497478806974146

0.5521621579516159 0.7231626677654279 0.6502521193025917

0.5592221111699466 0.2039513358262207 0.7690168178637025

0.4407778888300509 0.7960486641737774 0.2309831821362933

0.9172262593663432 0.2201390644756466 0.4163465931291362

0.0827737406336676 0.7798609355243443 0.5836534068708719

0.7511241226877797 0.4660530227084571 0.9736184070135298

0.2488758773122292 0.5339469772915475 0.0263815929864738

0.6297289541046271 0.1781987529863039 0.6440539874625580

0.3702710458953727 0.8218012470136894 0.3559460125374495

0.6155102834005981 0.3144387057005462 0.7855367218468485

0.3844897165994046 0.6855612942994601 0.2144632781531434
